# Editorial: Health Disparities—An Important Public Health Policy Concern

**DOI:** 10.3389/fpubh.2017.00099

**Published:** 2017-05-03

**Authors:** Haichang Xin

**Affiliations:** ^1^University of Alabama at Birmingham, Birmingham, AL, USA

**Keywords:** health disparities, public health, policy, socioeconomic status, systematic approach

**Editorial on the Research Topic**

**Health Disparities—An Important Public Health Policy Concern**

Health disparities are an important public health policy concern as they are related to the social inequalities in population health. Health disparities are not just a problem for the persons experiencing them but also a concern for the entire population in the society. Take the U.S. as an example, there are large health status gaps between racial groups, and the health of the racial minorities in the U.S. will negatively impact the overall health of the nation. Health disparities are also costly and particularly burdensome to the U.S. health-care system. A 2009 report on the economic burden of health disparities in the U.S., commissioned by the Joint Center for Political and Economic Studies, revealed that between 2003 and 2006, an estimated 30.6% of direct medical care expenditures for African-Americans, Asian-Americans, and Hispanics were excess costs due to health inequalities (1). African-Americans, Hispanics, and Asians accounted for $229.4 billion in direct medical expenditures due to health disparities (1). It is clear that health disparities are prevalent in the health-care system.

As such, the *Frontier in Public Health Epidemiology* journal has organized a special topic on health disparities, as it has become an important public health policy concern. This edition has called for papers to explore reasons at different levels and angles and suggest promising interventions to reduce these disparities. All the submissions have gone through the peer review process. By the time of submission closing, we have accepted seven articles for publication.

These papers cover a wide range of topics within health disparities, including obesity-related behaviors among poor adolescents and young adults, the association between prevention of human papillomavirus infection and malignancies, residential segregation and overweight/obesity, hopelessness and depression, education and alcohol consumption, stressful life events and depression, and depressive symptoms and all-cause mortality. Specifically, these studies have examined how demographic factors, such as gender, race, and socioeconomic status, play a role in these topics. Socioeconomic status measured in these studies includes parental education and household expenditures, community and society subjective social status, school dropout status, segregated neighborhoods with paucity of parks and recreational facilities, prevalence of fast food outlets, dearth of healthy food options, and dangerousness that discourages outdoor activities.

These studies have used different data, such as national survey data and systematic review of literature. These demographic and socioeconomic factors have been measured at the individual level, household level, and community level, which contributes to academic discussion by understanding how these various levels of factors independently and jointly affect health disparities.

In fact, these studies support the theory of social determinants of health (2), which proposes that health status is jointly determined by factors at different levels and angles. The first factor is the physical and socioeconomic environment, such as quality of water, air, soil, urban rural locations, education, and poverty levels. The second factor is the living style and behaviors, such as levels of physical exercises, positive or negative mood, diet, smoking, and alcohol use. The third factor is the genetics, and the last factor is the medical care systems, such as preventive care and tertiary care.

These studies inform policies that meaningful interventions to reducing health disparities may be a systematic comprehensive social engineering process. Any individual stakeholder alone, such as communities, individuals, and health systems, may not be sufficient to address this comprehensive problem of health disparities. This social engineering process requires concerted efforts among multiple stakeholders over a long time that calls for changes in the physical and socioeconomic environment, living style and behaviors, and medical care organization and delivery.

We have discussed this topic “Health disparities—an important public health policy concern” in a broad context in an aim to increase the awareness of the importance, comprehensiveness, and promise of this topic.

## Author Contributions

HX has drafted this editorial.

## Conflict of Interest Statement

The author declares that the research was conducted in the absence of any commercial or financial relationships that could be construed as a potential conflict of interest.
